# The physiological basis for individualized oxygenation targets in critically ill patients with circulatory shock

**DOI:** 10.1186/s40635-024-00651-6

**Published:** 2024-08-22

**Authors:** Anne-Aylin Sigg, Vanja Zivkovic, Jan Bartussek, Reto A. Schuepbach, Can Ince, Matthias P. Hilty

**Affiliations:** 1 Institute of Intensive Care Medicine, University Hospital of Zurich, Raemistrasse 100, 8091 Zurich, Switzerland; 2 Department of Intensive Care, Erasmus MC, University Medical Center, Rotterdam, The Netherlands

**Keywords:** Microcirculation, Critical care, Tissue oxygenation, Tissue perfusion, Blood transfusion, Circulatory shock, Oxygen, Resuscitation, Hypoxia, Hyperoxia

## Abstract

**Background:**

Circulatory shock, defined as decreased tissue perfusion, leading to inadequate oxygen delivery to meet cellular metabolic demands, remains a common condition with high morbidity and mortality. Rapid restitution and restoration of adequate tissue perfusion are the main treatment goals. To achieve this, current hemodynamic strategies focus on adjusting global physiological variables such as cardiac output (CO), hemoglobin (Hb) concentration, and arterial hemoglobin oxygen saturation (SaO_2_). However, it remains a challenge to identify optimal targets for these global variables that best support microcirculatory function. Weighting up the risks and benefits is especially difficult for choosing the amount of oxygen supplementation in critically ill patients. This review assesses the physiological basis for oxygen delivery to the tissue and provides an overview of the relevant literature to emphasize the importance of considering risks and benefits and support decision making at the bedside.

**Physiological premises:**

Oxygen must reach the tissue to enable oxidative phosphorylation. The human body timely detects hypoxia via different mechanisms aiming to maintain adequate tissue oxygenation. In contrast to the pulmonary circulation, where the main response to hypoxia is arteriolar vasoconstriction, the regulatory mechanisms of the systemic circulation aim to optimize oxygen availability in the tissues. This is achieved by increasing the capillary density in the microcirculation and the capillary hematocrit thereby increasing the capacity of oxygen diffusion from the red blood cells to the tissue. Hyperoxia, on the other hand, is associated with oxygen radical production, promoting cell death.

**Current state of research:**

Clinical trials in critically ill patients have primarily focused on comparing macrocirculatory endpoints and outcomes based on stroke volume and oxygenation targets. Some earlier studies have indicated potential benefits of conservative oxygenation. Recent trials show contradictory results regarding mortality, organ dysfunction, and ventilatory-free days. Empirical studies comparing various targets for SaO_2,_ or partial pressure of oxygen indicate a U-shaped curve balancing positive and negative effects of oxygen supplementation.

**Conclusion and future directions:**

To optimize risk–benefit ratio of resuscitation measures in critically ill patients with circulatory shock in addition to individual targets for CO and Hb concentration, a primary aim should be to restore tissue perfusion and avoid hyperoxia. In the future, an individualized approach with microcirculatory targets will become increasingly relevant. Further studies are needed to define optimal targets.

## Introduction

**Fig. 1:**
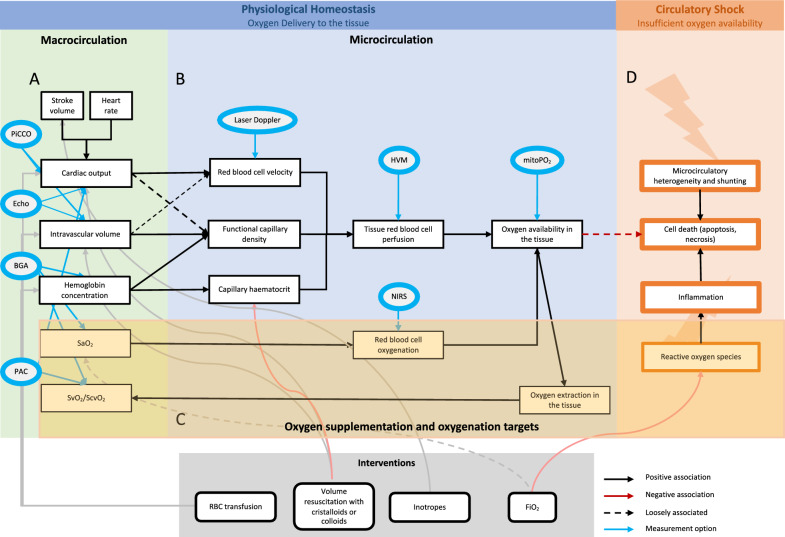
Overview of tissue oxygenation. Physiological homeostasis of oxygen delivery to the tissue depends on macrocirculatory (**A**) and microcirculatory (**B**) parameters. The macrocirculatory parameters, such as Hb concentration, SaO_2_, CO and intravascular volume rely on the microcirculatory function. Circulatory shock (**D**) with insufficient oxygen availability in the tissue is due to reactive oxygen species, inflammation and microcirculatory heterogeneity leading to cell death. The review aims to focus on oxygenation targets, representing a delicate balance between risks and benefits (**C**). Interventions to influence the different parameters are shown in grey. The measurements options are shown in blue. *PiCCO* Pulse Contour Cardiac Output, *Echo* Echocardiography, *BGA* Blood Gas Analysis, *PAC* Pulmonary Artery Catheter, *HVM* Hand-held Vital Microscopy, *mitPO*_*2*_ mitochondrial PO_2_, *NIRS* Near-Infrared Spectroscopy

Circulatory shock, which is defined as a life-threatening state of circulatory system failure associated with decreased tissue perfusion, leading to inadequate oxygen delivery (DO_2_) to meet cellular metabolic demands, remains a common condition with high morbidity and mortality in the intensive care unit (ICU) [1, 2]. Rapid restitution and maintenance of adequate tissue perfusion and oxygenation is the main treatment goal in critically ill patients in shock [3]. The determinants of global DO_2_ are the cardiac output (CO), the hemoglobin (Hb) concentration and the oxygen saturation in the arterial blood (SaO_2_). Hemodynamic management in the ICU thus aims to optimize these three physiological variables (Fig. 1A). However, defining targets for each of these variables to rapidly restore tissue perfusion while avoiding adverse effects associated with over-resuscitation (Fig. 1D), remains a challenge.

Current resuscitation protocols often emphasize an increase in CO. Interventions are guided by volume or inotrope responsiveness of the stroke volume (SV), a concept based on the Frank-Starling relationship [4, 5] and contractility. They are implemented in various ways in clinical practice [6–8]. The focus of the resuscitation is primarily the macrocirculation (Fig. 1A) although in circulatory shock the coherence between the macro- and the microcirculation is often uncoupled. This is shown by an absence of increase in tissue perfusion even though SV might still be responsive to a hemodynamic intervention. Thus, even in presence of persistent fluid responsiveness continued volume resuscitation may be associated with a negative effect on tissue perfusion [9] and with worse outcome [10, 11]. In the case of volume resuscitation, this can be partly explained by a reduction in tissue perfusion with red blood cells through hemodilution related decrease in capillary hematocrit. Without knowledge of the determinants of tissue perfusion and oxygenation, the optimal target for CO remains unknown and may vary from person to person [12]. Additionally, the relationship between DO_2_ and consumption in sepsis and septic shock has been found to depend on the presence of microcirculatory shunting in addition to mitochondrial dysfunction [13]. Recent technological developments that allow direct bedside assessment of microcirculatory function could open up the possibility of targeting microcirculation [14–16] and put individualized, tissue red blood cell perfusion focused therapy within reach [17, 18].

The second determinant of global DO_2_, Hb concentration, directly facilitates oxygen transport in the blood as oxygen is very poorly soluble in blood plasma (< 3%). Anemia due to various causes is common in critically ill patients. However the transfusion thresholds for these patients are mainly based on two trials, the TRICC [19] and TRISS [20] trial. The trials showed a similar 90-day mortality comparing a Hb of 7 g per deciliter (g/dl) and of 9 g/dl. In patients with septic shock, mortality at 90 days, rates of ischemic events and use of life support were similar in those with a higher Hb target and those assigned to blood transfusion at a lower threshold; the latter group received fewer transfusions [20]. Following these trials Hb targets between 7 and 8 g/dl were defined for most patients, depending on some general additional factors, such as hemodynamic instability, acute bleeding, or risk factors such as previous surgery or coronary artery conditions [21–23]. However, these studies do not fully represent the heterogeneous population of critically ill patients suffering from different types of circulatory shock. Nevertheless, the commonly used Hb concentration targets provide little individualization and often do not consider its role in the restoration of tissue perfusion and organ function (e.g., kidney) in patients with circulatory shock [24].

In terms of optimizing the risk–benefit ratio of hemodynamic stabilization of patients with circulatory shock, oxygen supplementation to increase oxygen content per blood volume, in absence of lung disease, may be the most important to consider (Fig. 1C). A stronger focus on the risks associated with the intervention is desirable, because on the one hand, changes in blood oxygenation within the physiological range of oxygen saturation, according to the dissociation curve, only marginally influence global oxygen supply. On the other hand, supramaximal pulmonary and blood oxygenation can be associated with an increased potential for negative effects. However, increasing acidosis due to tissue hypoperfusion may result in increased DO_2_ due to the Bohr effect on the dissociation curve. Previous studies have demonstrated that critically ill patients often show high SaO_2_ values even though there are indications that the relationship between SaO_2_ and mortality likely is U-shaped [25]. The difficulty in defining SaO_2_ targets may thus represent a risk for hyperoxia based on fear of hypoxia and avoiding hyperoxia could represent a promising strategy to improve patient management.

This narrative review aims to explore the factors influencing decision-making regarding oxygenation targets in critically ill patients with circulatory shock. It examines the risks and benefits of oxygen supplementation by assessing the physiological basis for DO2 and the regulatory mechanisms designed to counteract deficiencies. By providing an overview of the relevant literature, we aim to support decision making at the bedside and provide an outlook on future trends.

### Oxygen delivery to the tissues is the basis for all processes of life

To reach the current understanding of the role of oxygen in sustaining life has taken many centuries of research. Oxygen is essential for modern metazoan organisms, which emerged around 300 million years ago, coinciding with the significant rise of oxygen levels in Earth’s atmosphere [26]. Oxygen was independently discovered by the English chemist Joseph Priestley and Carl Wilhelm Scheel around 1774 [27], and was named by Antoine Lavoisier in 1778. The “Pneumatic Institution”, founded 1798 in Bristol, was one of the first places where the effects of oxygen on the human organism were examined in the setting of different illnesses. In collaboration with James Watt and Humphry Davy many new methods to deliver oxygen to patients were developed. The research was accelerated at the beginning of the twentieth century with the discovery of oxygen tensions as partial pressure by Adolf Fick and Paul Bert. But it was not until 1917 that John Scott Haldane, following a coal mine explosion, developed the first face mask with a possibility to adjust the administration of oxygen [28]. However, the administration of supplemental oxygen is only the first step, as the oxygen must find its way to the tissue, where oxidative phosphorylation takes place. Oxygen rich blood travels through a network of branching vasculature and is distributed in the tissue by the microcirculation, consisting of arterioles, capillaries, and post-capillary venules with a diameter below 20 µm. The red blood cells, which measure between 3 and 6 µm, travel through the capillaries in a single file fashion and provide oxygen via convection and diffusion [15]. The former occurs through the movement of Hb-bound oxygen molecules from the red blood cells in the capillary network to the mitochondria to fulfil their metabolic function [29]. In this process, the high affinity of the cytochrome *c* oxidase, the enzyme that reduces oxygen to water, to oxygen plays an important role in maintaining homeostasis by binding oxygen over a wide range of local oxygen pressures in the mitochondria, as low as 0.3–1.0 kPa. This remarkable property forms the basis for the oxygen conformance theory, which states that only at the extremely low end of tissue oxygenation, oxygen demand becomes dependent on supply. In other words, the functionality of oxidative phosphorylation as the basis of all life, can be maintained in the most extreme of conditions [30, 31].

### Physiologic adaptation to hypoxemia demonstrates the adaptability of the pulmonary and systemic microcirculation

In line with the importance of maintaining oxidative phosphorylation, the physiological processes along the oxygen supply chain are aimed at avoiding hypoxemia and hypoxia, the former referring to low blood oxygen content, and the latter, to low oxygen levels in the tissue. Genetic and physiological adaptation mechanisms to hypoxia ensure the maintenance of the homeostasis in states of external limitation of oxygen supply, and internal causes of tissue mal perfusion due to systemic disease. However, before understanding the role of hypoxia in disease, isolated models of tissue hypoxia were needed to examine these intrinsic mechanisms. Early research on adaptation to hypoxia was performed by Paul Bert in his compression chamber at the University of Sorbonne in Paris in the nineteenth century. In the following twentieth century subsequent field research was extended to high altitude locations around the world [32]. As partial pressure of oxygen decreases with ascent to high altitudes, the human body relies on an intricate system to detect the lower oxygen availability and react to it to maintain adequate tissue oxygenation. Some of these mechanisms focus on the functioning of the lungs, others on the systemic organs. In general, all animals express hypoxia-inducible factor (HIF) 1, composed of HIF-1α and HIF-1β, and vertebrates also produce HIF-2 and HIF-3. HIF-1 and HIF-2 can activate gene transcription which in turn regulates systemic DO_2_ and utilization, the role of HIF-3 is less well known. HIF-1 is regulated by oxygen-dependent hydroxylation by the von Hippel-Lindau protein. The O_2_-dependent binding is inhibited during hypoxic conditions and the HIF-1 activates some and inhibits other genes. At the tissue level, hypoxia leads to angiogenesis via the regulation of vascular endothelial growth factor and to a shift to anaerobic metabolism via the induction of glycolysis and glucose transporters. At the same time HIF-2 regulates several genes that control erythropoiesis [33]. Moreover, HIF are crucial in a multitude of mechanisms protecting cells from oxidative stress by increasing antioxidant production and decreasing oxidant production [34]. While HIF effectively regulates medium- and long-term responses on a cellular level, immediate physiological adaptation is needed to provide acute adaptation to hypoxia.

**Fig. 2:**
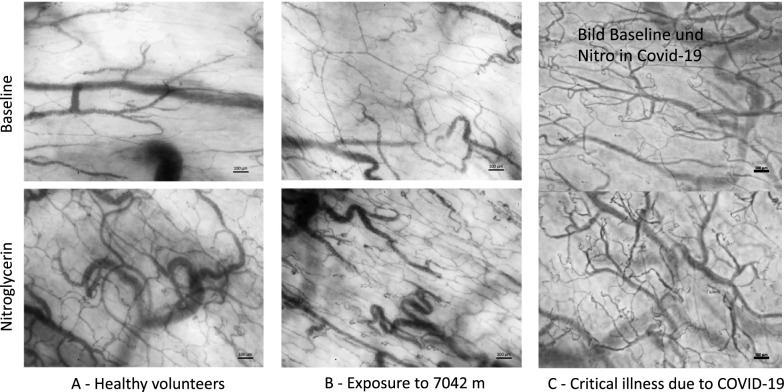
Sublingual microcirculation images. Representative images of the sublingual microcirculation before and after the topical application of nitroglycerin, during exposure to extreme altitude, and in critically ill COVID-19 patients. The sublingual microcirculation shows a similarly reaction to hypoxia in healthy volunteers at high altitudes, and critically ill COVID-19 patients. The application of a topical nitroglycerin in healthy volunteers leads to an increase of capillary density that is similar to adaptation to high altitude. Adapted from [42, 45]

In order to regulate the function of the cardio-respiratory system during hypoxia, oxygen levels are sensed rapidly at the glomus caroticum, which is located at the bifurcation of the internal and external carotid arteries. The chemoreceptor tissue, which contains type I neuronal glomus cells and type II sustentacular, glia-like cells, is sensory innervated by the carotid sinus nerve. The exact mechanism to detect hypoxia in these cells is not yet found and still under debate. It is assumed that hypoxia depolarizes the glomus cells through a inhibition of K + cannels and that the subsequent calcium-dependent release of excitatory neurotransmitters increases the neuronal activity [35]. In this way, cardiovascular and respiratory responses are triggered and / or modulated. In addition, different parts of the circulatory system have intrinsic regulation mechanisms. The pulmonary circulation responds with vasoconstriction of the pre-alveolar arterioles to a decrease of alveolar oxygen partial pressure. The effect was first described by Bradford and Dean in 1889 and was subsequently named Euler–Liljestrand-reflex [36]. Its rapid onset results from constriction of the small intrapulmonary arteries, mainly the pre-capillary vessels but also, to some extent, the post-capillary venules [37]. The sensory mechanism to detect alveolar hypoxia seems to be within the mitochondria of the smooth muscle cells of the pulmonary arteries [38]. Thanks to this mechanism, a ventilation-perfusion mismatch can be avoided. In global hypoxia, such as at high altitude or with diffuse lung damage, a diffuse Euler-Liljestrand-reflex leads to an increase of pulmonary artery pressure [39]. In the systemic circulation, on the other hand, the focus is to optimize oxygen availability in the tissues (Fig. 1B). Autoregulation of arterial tone plays an important role in the regional distribution of blood flow [40]. An increase in the activity of the sympathetic nervous system during acute hypoxemia, and above all a reduction of the activity of the parasympathetic nervous system in the following weeks, appears to be responsible for an increase in heart rate [41]. Simultaneously changes in plasma volume appear to cause a decrease in SV which ultimately leads to a constant CO [32]. These changes are often confounded by additional factors such as exercise or hypovolemia. Furthermore, systemic vascular tone and systemic vascular hindrance have been found to remain unaffected during ascent to high altitude. Recent observations have led to a deeper understanding of the mechanisms to increase DO_2_ to the tissue during hypoxic exposure. In a large study of healthy volunteers ascending to 7124 m, recruitment of pre-existing capillaries was identified as the main physiological response to increase microcirculatory oxygen extraction capacity at high altitude [42]. A variability in the response of the microcirculation has been described in different organs [43, 44]. Dark field microscopy images of the sublingual microcirculation recorded in healthy volunteers at sea level and after 2 weeks at 7042 m, representative for the response mechanisms to hypoxia, are shown in Fig. 2A, B.

### Effects and adaptation to hypoxemia in critically ill patients

Different to volunteers at high altitude, critically ill patients in circulatory shock often present with insufficient tissue oxygenation due to impaired microcirculation. In sepsis and septic shock, the microcirculatory alterations also include primary damage to the microcirculation caused by the inflammatory processes and changes to the coagulation system, resulting in a reduced functional capillary density, more non-perfused and intermittently perfused capillaries and an increase in perfusion heterogeneity [3] (Fig. 1D). Other forms of circulatory shock can lead to similar alterations due to secondary damage to the endothelial cells and the tissue [18]. In critically ill patients, altered microcirculation without improvement in disease progression has been shown to be a strong predictor for poor outcome with higher mortality [46]. Measurement of microcirculatory function in critically ill patients with severe hypoxemia and higher SOFA scores due to COVID-19 ARDS showed increased microcirculatory diffusion and convection capacity this in contrast to other viral disease [45, 47, 48]. Representative dark-field microscopy of this population is shown alongside healthy volunteers adapted to high altitude in Fig. 2C. In these patients with isolated lung failure, it was thus possible to study the effects of hypoxemia on an otherwise functionally intact systemic microcirculation and it was shown that adaptation mechanisms to tissue hypoxia are similar to the adaption of healthy volunteers at high altitude. These findings confirm a physiological link between high altitude physiology and critical illness, where in both conditions tissue hypoxia is present. Furthermore, experimental data indicate protective effects associated with adaptation to hypoxia in states of disease, such as a reduced myocardial infarction size in mice when subjected to continuous normobaric hypoxia [49, 50]. These effects show that the intrinsic mechanisms of microcirculation can help the tissue to cope with hypoxemia, provided a sufficient global blood flow and availability of Hb as oxygen carrier.

### Hyperoxia may promote microcirculatory dysfunction and cell death through reactive oxygen species (ROS) production

In contrast to hypoxemia, hyperoxia, defined as excess of oxygen in the tissue and hyperoxemia, being a high blood oxygen content, are often caused by medical staff administering an overabundance of oxygen to the patient. Compared to the macro- and microhemodynamic effects of CO and Hb availability, the effect of differences in oxygen saturation achieved by oxygen supplementation is more difficult to quantify. Hyperoxia induced in the clinical setting by lack of awareness [51] can harm patients through production of ROS and induction of inflammation. At the time of discovery, Joseph Priestley was already discussing possible negative effects of oxygen. Shortly thereafter, Antoine Lavoisier discovered the presence of lung damage in guinea pigs after inhalation of pure oxygen [52]. In 1958, a first report was published on lung damage in humans detected after and possibly related to long-term oxygen therapy [53]. Later research located the main source of ROS within the respiratory chain of the mitochondria in the pulmonary vascular endothelial cells, where the precursor superoxide anion originates at complex III at the inner membrane of mitochondria. The superoxide anion in turn changes into hydrogen peroxide and further turns into water or hydroxyl radicals, which are the main ROS [54]. They are responsible for the adverse effects in tissues across the body. The primary effects of hyperoxia in the lung occur in the form of damage to pulmonary capillary endothelial cells, followed by destruction of pulmonary epithelial cells. Hyperoxia and associated high levels of ROS destroy cellular macro-molecules leading to cell death or initiating apoptosis (Fig. 1D). The effect on remote tissues depends on the inflammatory response with the secretion of chemo-attractants and pro-inflammatory cytokines attracting leukocytes. The leukocytes are thus indirect effectors and at the same time another source of ROS with consecutive inflammation and further destruction of lung and other tissue [55]. High levels of superoxide anions can lead to specific organ damage and ultimately, promote multi-organ failure [56]. The hyperoxic microcirculation primarily shows a decrease in capillary density, that may be accompanied by an increased heterogeneity of capillary perfusion as normally seen in septic patients [13, 57, 58]. Additionally, the mitochondrial oxygen tension (mitPO_2_) decreased over a level of 26.6 kPa PaO_2_ [59]. In the systemic vascular bed, hyperoxemia can increase vascular resistance and mean arterial pressure and may decrease CO [60, 61]. Despite this in ovine models of acute peritonitis hyperoxia lead to better macro- and microcirculatory parameters [62]. Whereas a systematic review of hyperoxia in sepsis and septic shock in humans showed in 6 out of 10 included studies an increased mortality [63]. A recent study with mechanically ventilated mice could show time- and dose-dependent immune response of hyperoxia with raised cytokines, neutrophils and chemokines [64]. Knowledge of the relationship between the fraction of inspired oxygen (FiO_2_) and the formation of ROS particularly above a threshold FiO_2_ of 0.6 [65], and the mechanisms leading to the adverse effects have increased awareness with oxygen supplementation.

### Lower versus higher oxygenation targets in critically ill patients

The recent advances in our understanding of the effects of both tissue hypoxia and hyperoxia, have underlined the importance of the level of oxygen supplementation not only in terms of a risk–benefit ratio in critically ill patients, but also because of potential protective effects of adaptation mechanisms to hypoxia. Based on the investigation of these pathophysiological mechanisms related to tissue oxygen availability, several clinical studies have been conducted in critically ill patients (Table 1). A trial published in 2014 compared different oxygen saturation (SpO_2_) targets (SpO_2_ 90–92% versus higher SpO_2_) and showed only a decrease in lactate levels but no other difference [66]. Another study comparing liberal targets SpO_2_ above 96% with a conservative group target (SpO_2_ 88–92%) pointed toward a slightly lower 90-mortality in the conservative group [67]. The Oxygen-ICU randomized clinical trial, published in 2016, showed lower ICU-mortality with less episodes of shock, liver failure and bacteremia in the conservative group with an SpO_2_ target of 94–97% (PaO_2_ 9.3–13.3 kPa) compared to the conventional group with SpO_2_ of 97–100% (PaO_2_ up to 20 kPa) [68]. The HYPERS2S-Trial was stopped prematurely when no benefit of hyperoxia with a FiO_2_ of 1.0 for 24 h compared to a conservative group with SpO_2_ 88–95% could be found [69]. The IOTA review and meta-analysis revealed a dose-dependent increased risk of short- and long-term mortality of patients treated with liberal oxygen [70]. The ICU-ROX investigators found no significant difference in mortality comparing a conservative group with SpO_2_ < 97% and an usual-oxygen group with no upper limits [71]. On the contrary the LOCO_2_ Trial was stopped early because of suspicion of an increased risk for serious adverse events and higher 90-day mortality in the conservative group [72]. The biggest prospective study of the HOT-ICU investigators comparing a lower-oxygenation group with PaO_2_ target of 8 kPa and a higher-oxygenation group with PaO_2_ of 12 kPa with a total of 2928 patients showed no difference in the 28-day mortality or serious adverse effects [73]. A post hoc subgroup analysis of the cohort did not show any difference in the 90-day mortality between the two groups [74]. Nevertheless, the lower oxygenation group had a significantly higher percentage of days alive without life support. Further a study from the Netherlands with 574 patients (low-normal group PaO_2_ 8–12 kPa, high-normal 14–18 kPa) also found no significant difference in organ dysfunction at 14 days, nor significant differences in 90-day mortality, duration of mechanical ventilation and ICU length of stay [75]. The US PILOT trial, involving 2541 patients, did now show any difference in the number of ventilatory-free days by day 28 between a lower (SpO_2_ 90%), intermediate (SpO_2_ 94%) and a higher (SpO_2_ 98%) oxygenation target group [76]. However, despite the set oxygenation targets, each group in the study experienced substantial periods of hyperoxia (SpO_2_ of 99–100%), accounting for 12.3% of the total measurements time in the lower group, 14.7% in the intermediate group, and 32.7% in the higher group. The ICONIC-trial, involving 664 patients, did not find any reduction of the 28-day mortality between a low-oxygenation target (PaO_2_ 6.6–10.6 kPa, SaO_2_ 91–94%) or a high-oxygenation target (PaO_2_ 14.6–20 kPa, SaO_2_ 96–100%) [77]. The recently published HOT-COVID-trial did show more days alive without life support at 90 days in the lower oxygenation group (PaO_2_ 8 kPa) compared to the higher oxygenation group (PaO_2_ 12 kPa) [78] but the mortality at 90 days did not differ between the two target groups. Furthermore a literature review with a meta-analysis of 16 trials could not point out a significant difference in mortality in higher or lower oxygenation target at maximum follow-up [79]. The effect of the distinct oxygenation targets in these studies are summarized in Supplementary Table 1. Currently there are two big pending studies, the UK-ROX trial with 16′500 patients and the MEGA-ROX trial with 40,000 patients.

**Table 1:** Study setting, comparisons and findings in the 12 original studies and the two meta-analyses on the effect of distinct oxygenation targets

Study	Author	Year	Setting and patient population	Comparison and findings	Mortality 28d	Mortality 90d	ICU mortality before 28d	Mortality 1 Year
Conservative oxygen therapy in mechanically ventilated patients: A pilot before-and-after trial	S. Suzuki, et al	2014	1 ICU, Australia. 105 patients. > 24 h MV	Conservative oxygen (SpO_2_ 90–92%, 54 patients) decrease in lactate levels as to conventional (SpO_2_ > 92%, 51 patients). No difference on any secondary outcome	OR 0.35 (0.12–1.06) *p* = 0.062 Nr of Events 16:9 (Conventional:conservative)			
Conservative versus liberal oxygenation targets for mechanically ventilated patients	R. Panwar, et al., for the CLOSE Study Investigators and the ANZICS Clinical Trials Group	2016	4 ICUs in Australia, New Zealand, France. 103 patients. < 24 h MV, expected ≥ 24 h MV	No difference between conservative (SpO_2_ 88–92%) or liberal group (SpO_2_ ≥ 96%) group Point estimate for 90-day mortality slightly lower in conservative group		Conservative 21/52 (40%) Liberal 19/51 (37%) *p* = 0.74 Adjusted HR 0.77 (0.40–1.50; *p* = 0.44)	Conservative 13/52 (25%) Liberal 12/51 (24%) *p* = 0.86	
Effect of conservative vs conventional oxygen therapy on mortality among patients in an intensive care unit. The Oxygen-ICU randomized clinical trial	M. Girardis, et al	2016	1 ICU, Italy. 434 patients. ARDS horowitz index > 150	ICU-mortality lower in conservative group (SpO_2_ 94–97%, PaO_2_ 9.3–13.3 kPa, 216 patients) vs conventional group (SpO_2_ 97–100%, PaO_2_ up to 20 kPa, 218 patients) (11.6 vs 20.2% *p* = 0.01) Less episodes of shock, liver failure and bacteremia in conversative group. Terminated early after earthquake with subsequent enrollment difficulties			Conservative 25/216 (11.6%) Conventional 44/218 (20.2%) Absolut RD 0.086 (0.017–0.150, *p* = 0.1)	
Hyperoxia and hypertonic saline in patients with septic shock (HYPERS2S): a two-by-two factorial, multicentre, randomised, clinical trial	P. Asfar, et al., for the HYPER2S Investigators and REVA research network	2017	22 ICUs, France. 434 patients. MV, septic shock, horowitz index > 100	Non-significant increase in mortality in hyperoxia group (FiO2 1.0 for 24 h and afterwards normoxia, 217 patients) with increase of 7.4% at day 28 and 6.4% increase at day 90 vs normoxia group (SpO_2_ 88–95%, 217 patients) Ended preliminary due to lack of any benefit in the hyperoxia and hypertonic saline group and the possible harm	Normoxia 77/217 (35%) Hyperoxia 93/217 (43%) *p* = 0.12 Isotonic saline 81/220 (37%) Hypertonic saline 89/214 (42%) *p* = 0.25	Normoxia 90/217 (41%) Hyperoxia 104/217 (48%) *p* = 0.16 Isotonic saline 96/220 (44%), Hypertonic saline 98/2149 98 (46%) *p* = 0.48		
Conservative oxygen therapy during mechanical ventilation in the ICU (ICU-ROX)	D. Mackle, et al., for the ICU-ROX Investigators and the Australian and New Zealand Intensive Care Society Clinical Trials Group	2020	21 ICUs, New Zealand, Australia. 965 patients. MV (or non-invasive ventilation) for at least to the next day, no more than 2 h since starting the ventilation before randomization	At day 28 no significant difference of ventilator-free days between conservative-oxygen group (SpO_2_ upper limit at 97%, no minimal oxygen border, 484 patients) and usual-oxygen group (no upper limits, 481 patients). No difference in 28- or 180-day mortality or survival. Subgroup analysis with patients with hypoxic-ischemic encephalopathy with more ventilator-free days and less death in the conservative-oxygen group		Conservative 166/479 (34.7%) Usual 156/480 (32.5%) Unadjusted OR 1.10 (0.84–1.44)		
Liberal or conservative oxygen therapy for acute respiratory distress syndrome (LOCO_2_)	L. Barrot, et al., for the LOCO_2_ Investigators and REVA Research Network	2020	13 ICUs, France. 205 patients. ARDS, intubation and MV within the last 12 h, horowitz ≤ 300. Exclusion criteria: long-term oxygen, non-invasive home-ventilation, cardiac arrest, traumatic brain injury and cranial hypertension	Stopped early because of risk of serious adverse events and futility At day 28 mortality not significant different in the groups, at day 90 mortality was significant higher in conservative group (PaO_2_ 7.3–9.3 kPa during the first 7 days of MV) vs liberal group (PaO_2_ 12–14 kPa,) (44.4% vs 30.4%). 5 mesenteric ischemia in conservative group	Conservative 34/99 (34.3%) 95% CI 25.0–43.7 liberal 27/102 (26.5%) 95% CI 17.9–35.0	Conservative 44/99 (44.4%) 95% CI 34.7–54.2 Liberal 31/102 (30.4%) 95% CI 21.5–39.3	Conservative 36/99 (36.4%), 95% CI 26.9–45.8 Liberal 27/102 (26.5%) 95% CI 17.9–35.0	
Lower or higher oxygenation targets for acute hypoxemic respiratory failure (HOT-ICU)	O.L. Schjørring, et al., for the HOT-ICU Investigators	2021	35 ICUs, Denmark, Switzerland, Finland, the Netherlands, Norway, the United Kingdom, Iceland. 2928 patients. Hypoxic respiratory failure, ≥ 10 l oxygen in an open system or ≥ 0.5% FiO2 in a closed system	No difference in 90-day mortality or serious adverse events between lower-oxygenation group (PaO_2_ 8 kPa, 1462 patients) or higher-oxygenation group (PaO_2_ 12 kPa, 1466 patients)		Lower-Oxygenation 618/1441 (42.9%) Higher-Oxygenation 613/1447 (42.4%) RR 1.02 (0.94 to 1.11) RD 0.63 (− 2.92 to 4.17, *p* = 0.64) Adjusted OR 1.06 (0.90 to 1.24) *p* = 0.50		
Long-term mortality and health-related quality of life of lower versus higher oxygenation targets in ICU patients with severe hypoxaemia	E. Crescioli, et al	2022	35 ICUs, Denmark, Switzerland, Finland, the Netherlands, Norway, the United Kingdom, Iceland. 2928 patients. Hypoxic respiratory failure, at least 10 l oxygen in open system or at least 0.5% FiO2 in closed system	Adult ICU patients with severe hypoxaemia. Lower oxygenation targets (PaO_2_ 8 kPa, 1462 patients) did not improve survival or HRQoL at 1 year as compared to a higher oxygenation target (PaO_2_ 12 kPa, 1466 patients) until maximal 90 days after randomization				Lower oxygenation 707/1442 (49%) Higher oxygenation 704/1445 (48.7%) Adjusted RR 1 (0.93–1.08) Adjusted RD 0.4 (−3.2 to 4) Adjusted OR 1.02 (0.88–1.18), *p* = 0.92
Oxygen-saturation targets for critically ill adults receiving mechanical ventilation (PiLOT)	M. Semler, et al., for the PILOT Investigators and the Pragmatic Critical Care Research Group	2022	1 ICU, US. 2541 patients. Non-pregnant	No difference in number of ventilator-free days between lower target (SpO_2_ 90%; SpO_2_ goal range: 88—92%), intermediate target (SpO_2_ 94%; SpO_2_ goal range: 92—96%) or higher target (SpO_2_ 98%; SpO_2_ goal range: 96 – 100%) group	Lower target group 281/808 (34.8%) Intermediate-target group 292/859 (34.0%) Higher-target group 290/874 (33.2%) *p* = 0.81			
Oxygenation targets in ICU patients with COVID-19: A post hoc subgroup analysis of the HOT-ICU trial	B.S. Rasmussen, et.al	2022	35 ICUs, Denmark, Switzerland, Finland, the Netherlands, Norway, the United Kingdom, Iceland. 110 patients. COVID-19 positive. Hypoxic respiratory failure, ≥ 10 l oxygen in an open system or ≥ 0.5% FiO_2_ in a closed system	No difference in 90-day mortality Days alive without life support significantly higher in lower oxygenation group (PaO_2_ 8 kPa) versus higher oxygenation group (PaO_2_ 12 kPa)		Lower oxygenation group 22/55 (40.7%) Higher oxygenation group 23/55 (41.8%) *p* = 0.91		
Conservative versus liberal oxygenation targets in intensive care unit patients (ICONIC)	L.Imeen van der Wal, et al., for the ICONIC investigators	2023	9 ICUs, Netherlands, Italy. 664 patients. MV, expected ventilation duration of at least 24 h	No reduction of 28-day mortality between low-oxygenation target (PaO_2_ 6.6–10.6 kPa, SaO_2_ 91–94%) or high-oxygenation target (PaO_2_ 14.6 -20 kPa, SaO_2_ 96–100%)	Low-oxygenation target 129/335 (38.5%) High-oxygenation target 114/329 (34.7%) *p* = 0.34			
Lower vs higher oxygenation target and days alive without life support in COVID-19 the HOT-COVID randomized clinical trial	F.M.Nielsen, et al., for the HOT-COVID Trial Group	2024	11 ICUs, Denmark, Switzerland, Norway, Iceland, Wales. 726 patients. Confirmed COVID-19 and severe hypoxemia. Expected to receive oxygen for at least 24 h	More days alive without support in 90 days in the lower oxygenation group (PaO_2_ 8 kPa) than in the higher oxygenation group (PaO_2_ 12 kPa)		Days alive without support: 80 days (lower oxygenation group) 77 days (higher oxygenation group) *p* = 0.009 Mortality at 90 days: Lower oxygenation group 106/351 (30.2%) Higher oxygenation group 120/346 (34.7%) *p* = 0.18		
Mortality and morbidity in acutely ill adults treated with liberal versus conservative oxygen therapy (IOTA): a systematic review and meta-analysis	D. Chu, et al	2018	Meta-analysis, 16′037 patients. 25 trials exclusion criteria: pregnancy, extracorporeal life support, chronic respiratory disease, psychiatric disease, hyperbaric oxygen therapy and elective surgery	Liberal oxygen group with a dose-dependent increased risk of short-term (in hospital and 30 day) and long-term (up to 1 year, median 3 months) mortality	Liberal 484/7546 Conservative 422/7507 *p* = 0.033 RR 1.14 (1.01–1.28)			Mortality at longest follow-up Liberal 828/7897 Conservative 749/7857 *p* = 0.044 RR 1.10 (1.00–1.20)
Higher versus lower fractions of inspired oxygen or targets of arterial oxygenation for adults admitted to the intensive care unit	T. Klitgaard, et al	2023	Review with literature search, 19 RCTs, 10′385 patients RCTs comparing higher versus lower FiO_2_ or targets of PaO_2_, SpO_2_ or SaO_2_					Meta-analysis of 16 trials indicated no significant difference in mortality in higher or lower oxygenation target at maximum follow-up RR 1.01, 95% CI 0.96–1.06

These clinical trials show that oxygenation targets might be an important determinant of outcome, but the balance between risks and benefits may lie close together. This leads to an even greater challenge to define targets for oxygenation. Further studies should focus on exploring oxygenation targets in subpopulations of critically ill patients.

### Integration of microcirculation measurements in resuscitation of critically ill patients

**Fig. 3:**
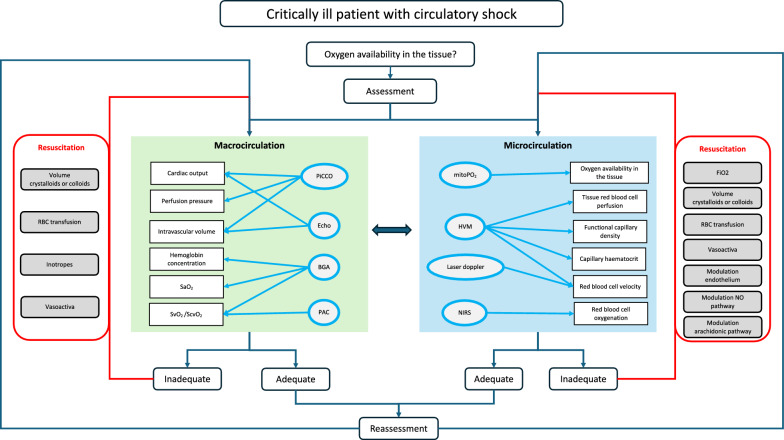
Resuscitation pathway. The oxygen availability in critically ill patients with circulatory shock should be assessed promptly. The macro- and microcirculation should be evaluated and addressed in parallel and the effect of resuscitation interventions should be re-assessed

Currently, the resuscitation of patients with circulatory shock is primarily focused on the macrocirculation. Tissue perfusion is restored by using crystalloids, inotropes, vasopressors and/or blood transfusions [8, 24]. For the primary assessment as well as the assessment of treatment response pulse contour analysis, the pulmonary artery catheter (PAC) and echocardiography are used. However, as the microcirculation determines the oxygen availability for the organs it should be assessed and restored in parallel to the macrocirculation [16] (Fig. 3). Bedside assessment of microcirculation is not well established today but there are different methods used in experimental settings which could also be used in the clinic. One promising option is the hand-held vital microscopy (HVM) that uses dark field imaging technique and can be performed sublingually. HVM enables to measure tissue red blood cell perfusion allowing differentiation of the effect of resuscitation measures on diffusion and convection capacity of oxygen carriers in the capillaries independently. New developments are underway to add two-wavelength measurements and ability to measure hemoglobin oxygen saturation in individual oxygen carriers as they move through the tissue. Another interesting tool is the non-invasively cellular oxygen metabolism measurement monitor (COMET), that measures mitochondrial oxygen tension (mitoPO_2_), being the real endpoint of the oxygen cascade. [59, 80]. Further, Near-Infrared Spectroscopy (NIRS) can give insight into red blood cell oxygenation and laser Doppler measures red blood cell velocity. It would be welcome if in the future there were a combined tool to measure the mitochondrial oxygen tension and other determinants of the microcirculation. The microcirculation can be influenced by conventional measures such as the manipulation of FiO2, the administration of fluids, RBC transfusions or vasoactiva but also modulation of the NO and the arachidonic pathways as well as the endothelium are discussed.﻿ Under certain conditions, the necessary measures for resuscitation of the macro- and microcirculation may be contradictory, for example a desired vasodilatation in the periphery with a need for vasopressors to maintain sufficient organ perfusion. It is important to develop appropriate schemes and test them in the clinic to determine appropriate cut-off values for determinates of the microcirculation. The goal would be a simple assessment of the microcirculation bedside with a corresponding algorithm for optimization.

## Conclusion

The three main determinants of the global DO_2_ are the CO, the Hb concentration and SaO_2_. Although it remains challenging to define targets for all three variables and these must be individually adjusted, the emerging literature shows that avoiding hyperoxia is essential to improve the risk–benefit ratio of hemodynamic stabilization in critically ill patients with circulatory shock. In absence of pulmonary disease, oxygen supplementation to increase SaO_2_ may be one of the least effective means to increase oxygen availability in the tissue. Limiting oxygen supplementation may provide a promising approach to reduce adverse effects of oxygen and even promote protective adaptation mechanisms. Advances in the direct measurement of tissue perfusion and mitochondrial oxygen tension could provide a novel approach to bring tissue-centric, individualized resuscitation at the bedside, increase awareness of the interplay of the SaO_2,_ the CO and the Hb concentration and improve the risk–benefit ratio of hemodynamic interventions.

For the definition of clear targets in critically ill patients further studies are needed. Based on the current literature, we recommend a conservative approach providing only the minimum necessary FiO_2_ to effectively prevent hyperoxemia and hyperoxia.

## Data Availability

Not applicable.
